# The effects of tetrahydrocannabinol and cannabidiol on sleep in cancer patients

**DOI:** 10.1007/s12094-025-04069-8

**Published:** 2025-10-21

**Authors:** Apoorva C. Reddy, John M. Hampton, Susan J. Park, Faith Dickerson, Betty Chewning, Natalie Schmitz, Kristine Kwekkeboom, Heather Neuman, Amy Trentham-Dietz

**Affiliations:** 1 Department of Surgery, School of Medicine and Public Health, University of Wisconsin-Madison, 750 Highland Avenue, Madison, WI 53726 USA; 2 Department of Population Health Sciences and Carbone Cancer Center, School of Medicine and Public Health, University of Wisconsin-Madison, Madison, USA; 3 Analytics Division, Minnesota Office of Cannabis Management, Saint Paul, USA; 4 School of Pharmacy, University of Wisconsin-Madison, Madison, USA; 5 School of Nursing, University of Wisconsin-Madison, Madison, USA

**Keywords:** Cancer, Cannabis, Sleep, Palliative care, Longitudinal studies

## Abstract

**Background:**

Despite limited research, cancer patients are opting for compounds found in cannabis, like tetrahydrocannabinol (THC) and cannabidiol (CBD), to improve their sleep. The purpose of this study was to examine the therapeutic value of cannabis for sleep.

**Methods:**

Patient-reported symptom responses were obtained from 1962 cancer patients enrolled in the Minnesota Medical Cannabis Program (MMCP) from 2015 to 2023. Multivariable logistic and linear regression models were used to evaluate the associations between changes in reported sleep disturbance scores and the dose of THC, the dose of CBD, and the cannabinoid ratio (THC:CBD). Logistic and linear regression models were adjusted for sex, age, race, ethnicity, body mass index, and MMCP enrollment fee category. Linear regression models were additionally adjusted for baseline sleep disturbance score.

**Results:**

Compared to the highest quintile category of CBD dose, lower dose quintiles were 29–35% less likely to be associated with at least a 30% improvement in sleep disturbance scores. Sleep disturbance scores improved by 1.87 points on a 0–10 ordinal scale for cancer patients with CBD doses in the top quintile, and approximately 1.5 points for doses in lower quintiles. THC and THC:CBD doses were not consistently related to changes in sleep disturbance scores.

**Conclusion:**

Higher CBD doses may be associated with clinically meaningful improvements in sleep in cancer patients enrolled in a medical cannabis program.

## Introduction

Cancer patients most commonly use cannabis to alleviate difficulty sleeping [1, 2]. Sleep, a cornerstone of human health, is a complex physiological process that is vital for cognitive functioning, memory consolidation, and overall well-being [3, 4]. When sleep is disturbed, it can affect both sleep quantity and quality, resulting in fatigue, mood disturbances, and difficulties in cognitive performance [5–10]. Disturbed sleep refers to conditions and situations where an individual has trouble getting adequate sleep, achieving restful sleep, or maintaining sleep throughout the night [11]. Types of disturbed sleep include insomnia, sleep apnea, parasomnias, circadian rhythm sleep disorders, and sleep-related movement disorders. Research suggests that cannabis may help resolve these sleep-related challenges due to its sedative effects [12], but the relationship between cannabis and sleep disturbance is still unclear [12–16].

Small qualitative and clinical studies suggest that users find cannabis effective in combating insomnia and promoting sleep [16, 17]. One recent study found that sleep disturbances were one of the most-improved symptoms among medical cannabis users after 6 months [18]. Phytocannabinoids like tetrahydrocannabinol (THC) and cannabidiol (CBD) interact with the endocannabinoid system, which is considered important in various physiological processes, including the sleep–wake cycle [15]. Cannabinoid (CB) receptors are known to exist in two main forms: CB1, which is predominantly expressed throughout the central nervous system, and CB2, which is expressed in the peripheral nervous system, predominantly in immune cells. CB1 receptors in the forebrain and pons are thought to activate the serotonergic system that regulates the sleep–wake cycle. Research suggests that cannabis, especially THC-dominant cultivars, can reduce sleep latency (the time it takes to fall asleep), increase slow-wave sleep (a phase of deep sleep crucial for physical renewal) [19], and is associated with same-day improvements in sleep [16]. Meanwhile, CBD has shown the potential to reduce anxiety and promote relaxation, indirectly supporting sleep [15, 20]. However, the relationship between cannabis and sleep is intricate and multifaceted.

While THC and CBD may offer benefits for certain individuals, their effects on sleep, particularly rapid eye movement (REM) sleep, can also have negative consequences, depending on dosage and individual conditions. For instance, research has indicated that THC can suppress REM sleep and REM density [15], which is believed to be important for emotional regulation and memory [21, 22]. While this might result in fewer dreams or nightmares, which could be beneficial for individuals with conditions like post-traumatic stress disorder (PTSD) [22], the suppression of REM sleep could lead to adverse cognitive and emotional effects over time. In addition, the effects of THC and CBD are thought to be dose-dependent, and higher doses may lead to counterproductive effects [15]. In fact, higher THC doses have been associated with increased sleep latency, more frequent awakenings (sleep fragmentation), and less restful sleep [15, 23]. This means that while THC may help people fall asleep faster or stay asleep longer, it does not equate to better quality sleep. Disrupted sleep architecture, especially reduced REM and increased nighttime awakenings, can contribute to cognitive impairment the next day, despite longer sleep duration. However, additional studies using objective sleep measures are needed to confirm these theorized effects of THC.

A broad range of perspectives on cannabinoid utility are also manifest in recent cancer-focused research and may be driven by differences in each study’s cannabis product characteristics (i.e., route of administration, dosage regimen, and cannabinoid content). Still, the consensus favors cannabis-derived therapies for cancer patients managing sleep [24]. A randomized trial of cancer patients found that nightly oral administration of oil products with either a 1:1 or a 4:1 ratio of THC-to-CBD for 12 weeks significantly improved sleep [25]. A randomized trial of patients with advanced cancer and opioid-refractory pain found that low-dose nabiximols (1:1 THC-to-CBD ratio) administered for 5 weeks (1–4 sprays per day) reduced sleep disruption [26]. Among cancer patients undergoing treatment, sleep issues were reported to be the most-improved symptom with cannabis use [27].

Understanding the effects of cannabis on disturbed sleep can shed light on potential therapeutic applications and risks associated with its use in cancer patients. To explore this, we conducted a longitudinal retrospective study aimed at exploring the association between cannabis use patterns and disturbed sleep among cancer patients, focusing on the change in reported sleep disturbance and examining the route of administration, dose of THC, dose of CBD, and ratio of THC-to-CBD. The results from this study will lay the groundwork for further research to identify whether specific cannabis formulations are associated with positive effects among cancer patients.

## Patients and methods

### Study setting

This study was conducted within the Minnesota Medical Cannabis Program (MMCP), which authorizes patients with specific qualifying conditions to receive cannabis products [28]. Patients eligible for enrollment were certified by healthcare practitioners based on medical criteria such as cancer-associated symptoms. Following certification, patients consulted with licensed dispensary pharmacists who recommended cannabis formulations tailored to individual needs, including product type, administration route, and dosage. A label was put on the product packaging with the pharmacist's instructions to help patients recall the recommended dosage and frequency. Patients completed a Patient Self-Evaluation (PSE) survey at each dispensary visit using an online registry. These surveys documented baseline symptoms and tracked symptom progression over time.

### Study population

The initial sample consisted of 6069 cancer patients registered in the MMCP between July 1, 2015, and June 9, 2023. Patients were included if they were at least 18 years old. Additionally, patients were required to have sufficient follow-up, defined as at least two visits after 30 days of enrollment in the program. We excluded patients who reported raw cannabis use due to existing limitations in the process for quantifying THC and CBD amounts. After excluding patients under 18 years of age (*n* = 139), patients with insufficient follow-up visits (*n* = 1414), patients using raw cannabis (*n* = 484), and patients with missing symptom data (*n* = 14), the final analytical sample included 1962 adult patients who remained in the program for at least 30 days and had complete data for analysis (Appendix Figure 2).

### Data collection

Data were obtained from patient enrollment records, PSE surveys, and dispensary transaction logs. Patient enrollment records documented demographic variables, including age, sex, height, weight, race, ethnicity, and eligibility for reduced program fees. Disturbed sleep symptom severity was assessed on each PSE using a 0–10 ordinal scale, where 0 indicated no symptoms and 10 indicated extreme severity over the preceding 24 h [29, 30]. This scale is an adaptation of the single-item Sleep Disturbance Numerical Rating Scale (SD NRS), which has been validated in other patient populations with conditions, such as atopic dermatitis and prurigo nodularis [31, 32].

Dispensary transaction data provided detailed information about product composition (THC and CBD content in milligrams), the number of units dispensed, and estimated days of use based on pharmacist guidance. Cannabis products were categorized by route of administration: inhalation, enteral (oral), oromucosal (sublingual), and topical. Raw cannabis products were excluded due to limitations in THC and CBD quantification.

### Statistical analysis

The primary outcome was the change in disturbed sleep severity scores. Cannabis use patterns were assessed based on the patient’s first dispensary transaction after an initial 30-day stabilization period during which the MMCP patients develop their cannabis product preferences [30, 33]. The baseline measure is the PSE disturbed sleep severity score at enrollment (assessed on an 11-point scale). We averaged each patient’s PSE disturbed sleep severity scores per patient post-30 days of enrollment for comparison to their baseline score. This was calculated by comparing the enrollment symptom score with the average score from all post-30-day visits (excluding the initial post-30-day visit used to define cannabinoid exposure). For consistency, only patients with at least two post-30-day visits were included. If a patient’s visits included gaps of 120 days or more, only the data collected before the gap were included to prevent misattributing symptom changes.

Descriptive statistics summarized demographic and cannabis use characteristics. For regression models, predictor variables of interest were THC and CBD dosage, THC:CBD ratio, and route of administration. Multivariable linear regression was used to predict changes in sleep severity scores. Logistic regression models estimated odds ratios and 95% confidence intervals of experiencing a clinically meaningful (defined as ≥ 30% based on a review by Dworkin et al. 2008) reduction in disturbed sleep [34]. To do this, a dichotomous categorical variable was created to show whether each patient achieved a 30% or greater reduction in disturbed sleep.

The analysis was guided by the Gelberg–Andersen Behavioral Model for Vulnerable Populations to examine how cannabis use characteristics influenced symptom changes over time [35]. Based on guidance from the Gelberg–Andersen model, logistic and linear regression models were adjusted for covariates including age, sex, race, ethnicity, body mass index (BMI), and enrollment fee status. Linear regression models were also adjusted for baseline sleep disturbance score, and values above the 95th percentile for THC and CBD were set at the respective 95th percentile value. Graphs were created from separate analyses for the predicted probability of achieving a 30% or greater improvement in disturbed sleep for average dose per day (mg) for THC and CBD using restricted cubic spline with knots at 5, 27.5, 50, 72.5, and 95 percentiles. Similar graphs were created for the predicted improvement in disturbed sleep. All analyses were conducted using SAS (SAS Institute, Inc.).

## Results

### Patient and cannabis use characteristics

In the study sample, the mean age was 57.4 years (SD: 13.9), with a nearly even split by sex (51% male, 49% female). A minority of participating patients had metastatic cancer (6%). Most patients identified as White (89.1%) and non-Hispanic (95.3%). Approximately 40% qualified for the reduced program fee, which was used in this study as a proxy for patient financial status. On average, patients remained in the program for over 10 months and completed a median of five dispensary visits. The mean BMI was 26.9 kg/m^2^ (SD: 6.3).

During their participation, 75% of cancer patients used enteral (oral) cannabis products, either alone or in combination with inhalation products. The average daily THC consumption was 32 mg (SD: 71; median: 17 mg), while average CBD intake was 14 mg (SD: 44; median: 3 mg). Products with lower THC content were most often associated with the enteral route. The median THC:CBD ratio across all products was 7.21.

### Disturbed sleep symptoms

At enrollment, the average severity score for disturbed sleep was 6.72 out of 10. The mean severity score after cannabis use (excluding the first 30 days of enrollment in the MMCP) was lower by 1.63 points, corresponding to an average 24.3% reduction in sleep symptom severity (Table 1).

**Table 1:** Sample characteristics of cancer patients in the Minnesota medical cannabis program (MMCP), 2015–2023 (*N* = 1962)

Age (years)	N	%
18–39	239	12.2
40–49	250	12.7
50–59	504	25.7
60–69	603	30.7
≥ 70	366	18.7
Race		
White	1748	89.1
Black	55	2.8
Asian	27	1.4
Native American	40	2.0
Other/Multiple	33	1.7
No answer/unknown	59	3.0
Ethnicity		
Not Hispanic	1870	95.3
Hispanic	38	1.9
No answer/unknown	54	2.8
Sex		
Female	961	49.0
Male	1001	51.0
Metastatic cancer		
No	1845	94
Yes	117	6
Number of visits post-day-30		
2	452	23.0
3	280	14.3
4	219	11.2
5	151	7.7
6 +	860	43.8
MMCP enrollment fee		
Reduced price ($50)	791	40.3
Full price ($200)	1171	59.7

Adjusted regression analyses revealed a significant relationship between cannabinoid dose and symptom improvement. Patients in the lower quintiles of THC and CBD intake were less likely to experience clinically meaningful improvements in sleep disturbance (defined as ≥ 30% reduction in disturbed sleep score) [34]. Specifically, each increase in THC dose quintile was associated with a 0.4% increase in the odds of achieving meaningful sleep improvement (p = 0.06; Table 2). A similar trend was found for CBD dose (p = 0.02). THC:CBD ratio was not associated with a meaningful change in self-reported disturbed sleep symptom score (Table 2).

**Table 2:** Odds ratios and 95% Confidence intervals for the association between THC dose, CBD dose, THC:CBD ratio, and improvement of 30% or greater in disturbed sleep

2a: Association between THC dose and disturbed sleep					
Average THC dose per day (mg)	Improvement in disturbed sleep score		Odds ratio^a^	95% CI^a^	*p* value^a,b^
	< 30%	≥ 30%			
Quintile 1: 0–7.82	242	150	0.79	0.58–1.08	0.14
Quintile 2: 7.83–14.24	250	143	0.70	0.52–0.94	0.02
Quintile 3: 14.25–23.17	217	175	0.98	0.73–1.32	0.90
Quintile 4: 23.18–41.20	224	169	0.71	0.53–0.95	0.73
Quintile 5: > 41.20	218	174	1 (ref.)		
Continuous (per 5 mg) ^c^			1.02	1.00–1.04	0.06

2b: Association between CBD dose and disturbed sleep					
Average CBD dose per day (mg)	Improvement in disturbed sleep score		Odds ratio^a^	95% CI^a^	*p* value^a,b^
	< 30%	≥ 30%			
Quintile 1: 0–0.36	230	162	0.79	0.59–1.05	0.10
Quintile 2: 0.37–1.81	244	149	0.71	0.53–0.95	0.05
Quintile 3: 1.82–4.58	250	142	0.65	0.48–0.86	0.03
Quintile 4: 4.58–14.32	221	172	0.87	0.66–1.16	0.35
Quintile 5: > 14.32	206	186	1 (ref.)		
Continuous (per 5 mg) ^c^			1.04	1.01–1.07	0.02

2c: Association between THC:CBD ratio and disturbed sleep					
THC:CBD ratio	Improvement in disturbed sleep score		Odds ratio^a^	95% CI^a^	*p* value^a,b^
	< 30%	≥ 30%			
Quintile 1: 0–1.11	227	165	1 (ref.)		
Quintile 2: 1.12–3.84	231	162	0.99	0.74–1.32	0.93
Quintile 3: 3.85–16.87	216	176	1.13	0.85–1.51	0.39
Quintile 4: 16.88–59.33	253	140	0.76	0.56–1.03	0.08
Quintile 5: > 59.33	224	168	0.98	0.73–1.31	0.88

*THC* tetrahydrocannabinol, *CBD* cannabidiol, *CI* confidence interval, *ref.* reference

^a^Adjusted for sex, age, race, ethnicity, BMI, and MMCP enrollment fee

^b^*P* values for quintile categories are relative to the reference quintile

^c^For continuous linear regression, values above the 95th percentile for THC and CBD were set at the 95th percentile value

**Fig. 1:**
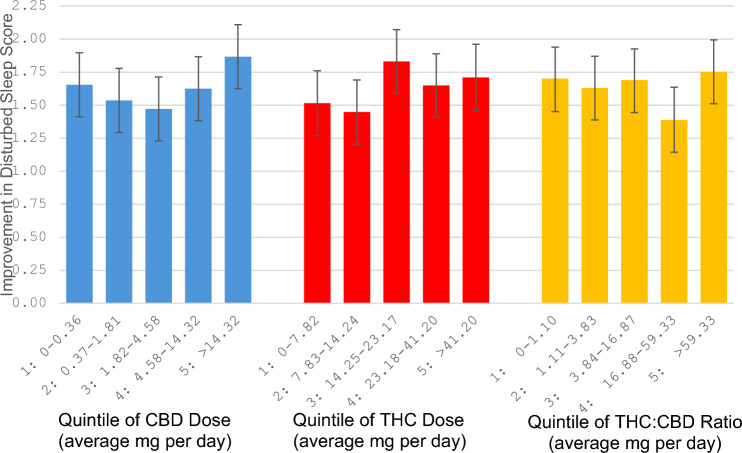
Quintile of CBD dose, THC dose, and THC-to-CBD ratio in relation to improvement in self-reported disturbed sleep score. The improvement in disturbed sleep score refers to the reduction in score from baseline to the average of disturbed sleep scores reported after 30 days of MMCP enrollment

**Fig. 3:**
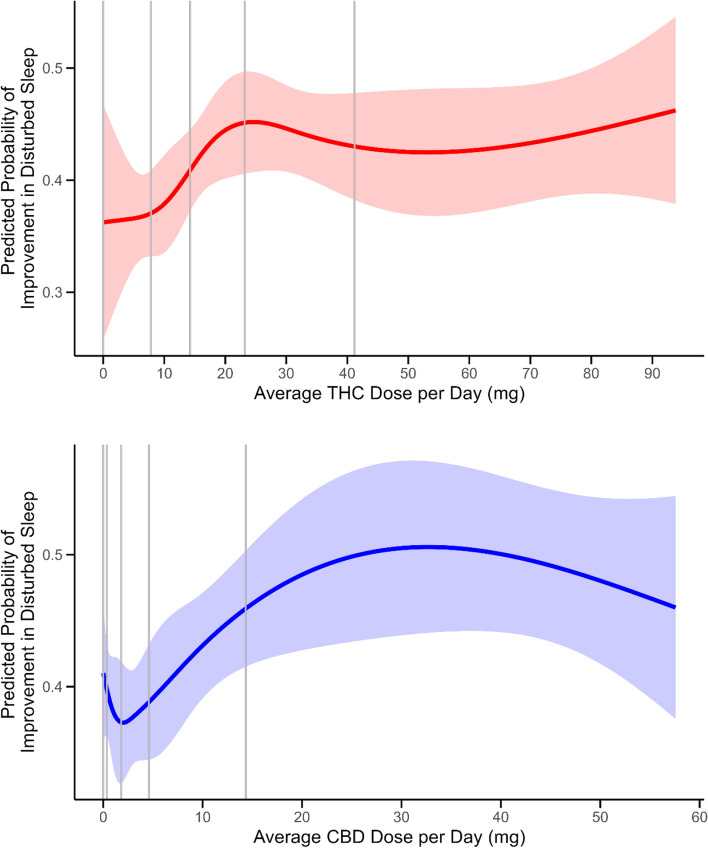
Predicted Probability of Achieving a 30% or Greater Improvement in Disturbed Sleep for Average Dose per Day (mg) for THC (red) and CBD (blue). Predicted probability using restricted cubic spline with knots at 5, 27.5, 50, 72.5, and 95 percentiles is shown with 95% confidence limits. Vertical gray lines mark quintile cut points for average dose

**Fig. 4:**
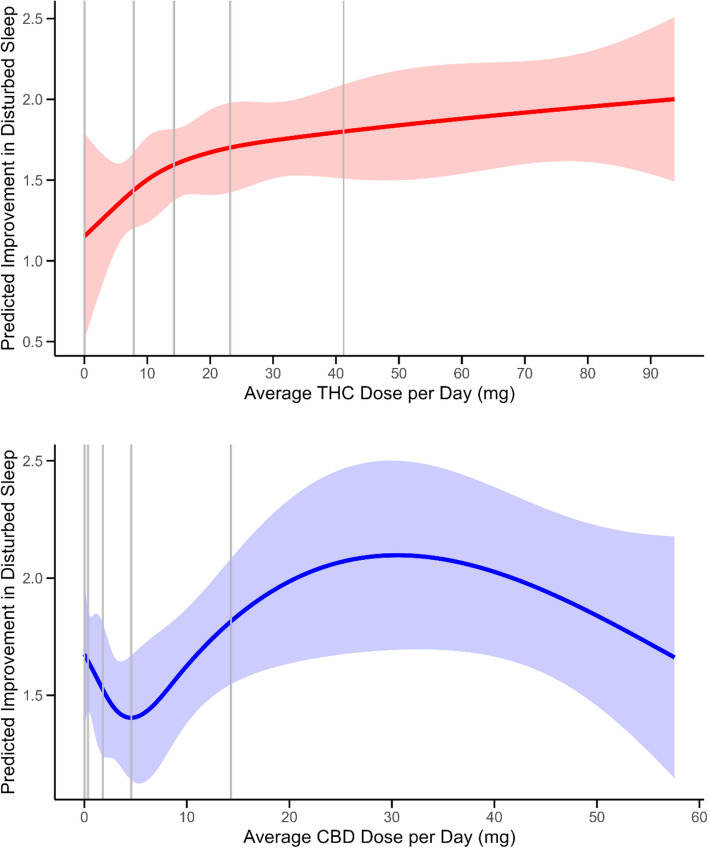
Predicted Improvement in Disturbed Sleep for Average Dose per Day (mg) for THC (red) and CBD (blue). Predicted values using restricted cubic spline with knots at 5, 27.5, 50, 72.5, and 95 percentiles are shown with 95% confidence limits. Vertical gray lines mark quintile cut points for average dose

When comparing predicted changes in disturbed sleep symptom scores across quintiles of THC dose, scores decreased more as dose increased, although the score changes were not meaningfully different from each other (p = 0.35, Table 3). Predicted changes in disturbed sleep symptoms according to quintile of CBD dose appeared U-shaped, with greater reductions in sleep disturbance scores for the highest and lowest doses (Fig. 1). Disturbed sleep symptom scores did not vary meaningfully according to THC-to-CBD ratio. Appendix 1, Figure 3 shows graphs of the predicted probability of achieving a 30% or greater improvement in disturbed sleep for average dose per day (mg) for THC and CBD using restricted cubic spline with knots at 5, 27.5, 50, 72.5, and 95 percentiles. Appendix 1, Figure 4 shows graphs of the predicted improvement in disturbed sleep.

**Table 3:** Predicted improvement in disturbed sleep vs. THC dose, CBD dose, and THC:CBD ratio

Average THC dose per day (mg)	Predicted improvement in disturbed sleep score^a^	95% CI^a^	p value^a,b^
Quintile 1: 0–7.82	1.52	1.27–1.76	0.29
Quintile 2: 7.83–14.24	1.45	1.21–1.69	0.15
Quintile 3: 14.25–23.17	1.83	1.59–2.07	0.50
Quintile 4: 23.18–41.20	1.65	1.41–1.89	0.73
Quintile 5: > 41.20	1.71	1.46–1.96	ref
Continuous (per 5 mg)^c^	0.011	−0.012–0.034	0.35
Average CBD dose per day (mg)	Predicted improvement in disturbed sleep score^a^	95% CI^a^	p value^a,b^
Quintile 1: 0–0.36	1.65	1.41–1.90	0.23
Quintile 2: 0.37–1.81	1.54	1.29–1.78	0.06
Quintile 3: 1.82–4.58	1.47	1.23–1.71	0.02
Quintile 4: 4.58–14.32	1.62	1.38–1.87	0.16
Quintile 5: > 14.32	1.87	1.62–2.11	ref
Continuous (per 5 mg) ^c^	0.038	0.003–0.074	0.04
THC:CBD ratio	Predicted improvement in disturbed sleep score^a^	95% CI^a^	p value^a,b^
Quintile 1: 0–1.11	1.70	1.45–1.94	ref
Quintile 2: 1.12–3.84	1.63	1.39–1.87	0.70
Quintile 3: 3.85–16.87	1.69	1.44–1.93	0.95
Quintile 4: 16.88–59.33	1.39	1.14–1.64	0.09
Quintile 5: > 59.33	1.75	1.51–1.99	0.75

*THC* tetrahydrocannabinol, *CBD* cannabidiol, *CI* confidence interval, *ref.* reference

^a^Adjusted for baseline symptom score, sex, age, race, ethnicity, BMI, and MMCP enrollment fee

^b^*P* values for quintile categories are relative to the reference quintile

^c^For continuous linear regression, values above the 95th percentile for THC and CBD were set at the 95th percentile value

No significant associations were observed between sleep symptom changes and the route of cannabis administration. Patients using various administration types, such as enteral, inhalation, oromucosal, and topical, showed similar patterns of disturbed sleep symptoms. The results from the analysis of routes of administration is included in Appendix 1, Table 4.

## Discussion

This study provides evidence that higher doses of THC and CBD may be associated with clinically meaningful improvements in disturbed sleep among cancer patients compared to lower doses. We examined these associations in two ways: by examining doses that may be more likely to be associated with a meaningful reduction in sleep disturbance, and by predicting changes in sleep disturbance symptom scores according to different doses. Results suggested that compared with lower dose quintile groups, THC doses in the fifth quintile group may be sufficient to reduce sleep disturbance symptoms by 30%, but that sleep disturbance scores are not statistically different from each other across dose quintiles. On the other hand, CBD doses in the fifth quintile group were more likely to result in greater symptom score reductions and a 30% reduction in scores compared with the 2nd and 3rd quintile groups. The Sleep Disturbance Numeric Rating Scale identifies a 2–4 point [31] and a 2–5 point [32] reduction on its 11-point scale as indicating a clinically meaningful improvement. The 1.87 point improvement for patients in the fifth quintile of CBD dose can be interpreted as an improvement approaching clinical significance, suggesting that CBD dosing plays a critical role in achieving clinically significant benefits. This is an important finding for the creation of dosage guidelines, which the MMCP does not currently provide. The only consistent MMCP recommendation to all patients is to start at a low dose and titrate up slowly. The rest of the dosing is specific to the patient and guided by consultations between the patient and pharmacist.

The results of this study are supported by the existing literature, where both THC and CBD are implicated in sleep quality [19]. THC was expected to generate a relatively rapid improvement in sleep that may last over 30 days, as seen in this study [16]. This study also confirms that higher doses of CBD result in greater sleep improvement [36, 37], even though, unlike THC, CBD is not thought to alter sleep architecture [38]. Studies have shown that low-to-moderate doses of CBD promote wakefulness in young adults and preclinical models [39, 40] and improve sleep in adults [41]. This study supports the possible age-dependent effects; low-dose CBD improved sleep after 30 days. Although THC-to-CBD ratio and route of administration did not impact disturbed sleep symptoms in this study, the observed individual dose-moderating effects of THC and CBD suggest a need for individualized dosing strategies to balance efficacy and potential side effects.

However, the effects of THC and CBD on REM sleep should be considered in the interpretation of these results. REM sleep plays a critical role in emotional regulation and memory consolidation, and its long-term disruption with THC use could exacerbate symptoms like mood disturbance or cognitive impairment [15, 21, 22, 42]. CBD, which is not believed to alter REM sleep significantly, may exert its effects on sleep through anxiolytic and anti-inflammatory mechanisms [15, 20]. In our study, CBD showed a more consistent dose–response relationship than THC, with higher doses associated with greater reductions in disturbed sleep symptoms. These findings support the hypothesis that CBD has more predictable sleep-promoting effects that may be moderated by stress, underlying health conditions, and concurrent medications.

Another critical consideration is the feasibility of using cannabinoids as a long-term treatment for sleep disturbance in cancer patients. Promisingly, a randomized trial of adults experiencing sleep disturbance found that chronic use of a low dose of CBD was safe and may improve sleep quality, but these effects do not surpass those of 5 mg melatonin [41]. If patients discontinue cannabinoid use after regularly using them, they may experience sleep disturbance associated with cannabis withdrawal [23]. Given that cannabis-based therapies are not covered by insurance in many settings, cost may be a barrier for some patients [43].

Limitations of this study include the use of cannabis transaction data as a proxy for cannabis product use and a lack of data on adherence. There was no formal assessment of patients' prior knowledge of cannabinoid dosing or non-MMCP experiences with cannabinoids, though this was typically discussed in patient consults with the dispensary pharmacist. Patients rated their sleep in the preceding 24 h, which may not accurately reflect their usual or actual sleep quality in comparison to more objective sleep measures, such as actigraphy and polysomnography. The time interval between cannabinoid dosing, sleep attempts, and sleep assessment was not captured either. Our analysis was limited by the lack of information on cancer type, stage of disease, and other treatments taken by patients, since these factors may have varying effects on sleep. Selection bias and reduced generalizability may have been introduced by the exclusion of patients who did not have at least two visits after 30 days of MMCP enrollment. The resulting sample was likely to be patients who were more optimistic about cannabinoid therapies and who were socioeconomically able to remain in the MMCP for a longer period. Finally, this study only included cancer patients from Minnesota who were predominantly White and non-Hispanic. This sample racial composition may restrict the generalizability of findings to more racially, ethnically, and geographically diverse populations.

Based on this study, there are many important aspects of sleep management with cannabis to further explore among cancer survivors. Future studies should incorporate objective sleep measures, such as polysomnography or actigraphy, to complement self-reported outcomes and further investigate the long-term effects of cannabinoid use on sleep [44]. Future studies should also collect data on previous cannabis use prior to medical cannabis program enrollment to determine whether habituation or tolerance occurs with continued use of cannabinoids for sleep. Additionally, studies should evaluate whether different cancer types and stages experience differences in reported benefits from cannabis use for disturbed sleep. Finally, there is a need to explore how the interplay between symptoms influences overall well-being and quality of life; cannabinoid dose changes may have downstream effects on other symptoms (e.g., depression and fatigue) that are linked to sleep disturbances in cancer patients [45].

Overall, this study suggests that the odds of achieving a clinically meaningful reduction in disturbed sleep symptom severity increase with higher doses of CBD. Questions remain regarding the role of THC dose in alleviating sleep disturbance and if very low levels of CBD are adequate for some patients, given the potential U-shaped relation between CBD and sleep symptom scores in this study. Healthcare providers can use these findings to better inform patients about the potential benefits of cannabinoid use for sleep disturbances.

## Figures and Tables

**Fig. 2:**
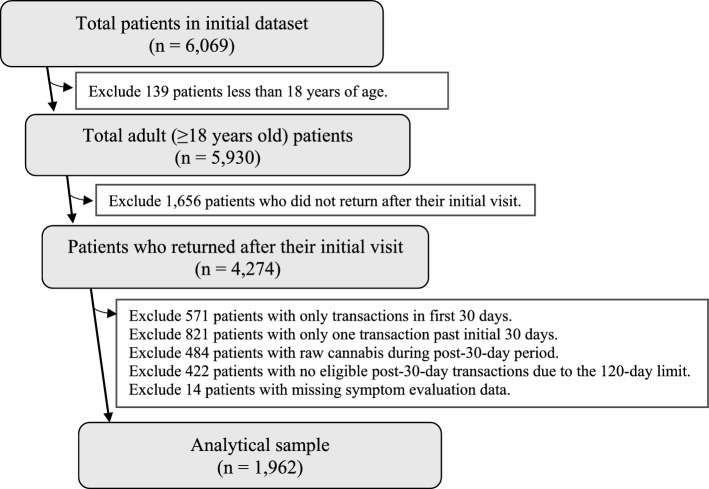
Flowchart of analytical sample selection process. Cannabis use patterns were assessed based on the patient’s first dispensary transaction after an initial 30-day stabilization period. Thus, patients were only included if they had two visits after that initial 30-day period of enrollment (one visit with a cannabis product purchase followed by a second visit with a symptom score evaluation). If a patient’s visits included gaps of 120 days or more, only the data collected before the gap were included to prevent misattributing symptom changes. The largest day supply for MMCP patients was 120 days. The 120-day limit prevented the misattribution of changes in symptom score to cannabis products or use

## Data Availability

The data supporting the findings of this study are available upon request from the corresponding author. The data are not publicly available due to privacy or ethical restrictions.

## Appendix

The products sold at the MMCP dispensaries were purchased through two manufacturers: RISE (Leafline Labs) and Green Goods (rebranded from Minnesota Medical Solutions and Vireo Health). Each manufacturer leads their own cultivation, production, and distribution of medical cannabis in Minnesota (1). Figure 2 below shows the flowchart of the sample selection for analysis.

See (Figs. 2, 3, 4 and Table 4

**Table 4:** Predicted improvement in self-reported disturbed sleep scores according to cannabis route of administration

Route of administration	N	%	Predicted sleep improvement (Score range: 0–10) ^a,d^	95% CI^a,d^	*p*-value^a,d^
Enteral					
None	487	24.8	1.54	1.29–1.79	Ref
Any	1475	75.2	1.67	1.51–1.83	0.35
Inhalation					
None	918	46.8	1.65	1.43–1.87	Ref
Any	1044	53.2	1.56	1.37–1.75	0.52
Oromucosal					
None	1368	69.7	1.61	1.43–1.79	Ref
Any	594	30.3	1.59	1.37–1.81	0.88
Topical					
None	1601	81.6	1.59	1.45–1.74	Ref
Any	361	18.4	1.61	1.35–1.88	0.89
Route Combinations					
Enteral only	483	24.6	1.69	1.46–1.91	Ref
Enteral + Inhalation	378	19.3	1.58	1.33–1.86	0.53
Inhalation only	289	14.7	1.63	1.39–1.93	0.79
Enteral + Oromucosal	197	10.0	1.88	1.54–2.23	0.34
Enteral + Inhalation + Oromucosal	119	6.1	1.39	0.94–1.83	0.25
Enteral + Inhalation + Topical	87	4.4	1.82	1.31–2.34	0.64
Enteral + Topical	83	4.2	1.58	1.05–2.11	0.72
Oromucosal only	76	3.9	1.44	0.89–1.99	0.42
Other combinations	250	12.7	1.52	1.22–1.82	0.39

*CI* confidence interval, *ref.* reference

^a^Adjusted for baseline symptom score, sex, age, race, ethnicity, BMI, and MMCP enrollment fee

^d^Adjusted for THC:CBD ratio

).
